# Polymorphism rs3742330 in microRNA Biogenesis Gene *DICER1* Is Associated with Pseudoexfoliation Glaucoma in Saudi Cohort

**DOI:** 10.3390/genes13030489

**Published:** 2022-03-10

**Authors:** Altaf A. Kondkar, Taif A. Azad, Tahira Sultan, Rakesh Radhakrishnan, Essam A. Osman, Faisal A. Almobarak, Glenn P. Lobo, Saleh A. Al-Obeidan

**Affiliations:** 1 Department of Ophthalmology, College of Medicine, King Saud University, Riyadh 12372, Saudi Arabia; mtanwar@ksu.edu.sa (T.A.A.); tasayed@ksu.edu.sa (T.S.); eosman@ksu.edu.sa (E.A.O.); falmobarak@ksu.edu.sa (F.A.A.); salobeidan@ksu.edu.sa (S.A.A.-O.); 2 Glaucoma Research Chair in Department of Ophthalmology, College of Medicine, King Saud University, Riyadh 12372, Saudi Arabia; 3 King Saud University Medical City, Department of Ophthalmology, King Saud University, Riyadh 12372, Saudi Arabia; 4 Department of Ophthalmology and Visual Neurosciences, University of Minnesota, Minneapolis, MN 55455, USA; rakeshr@umn.edu (R.R.); lobo0023@umn.edu (G.P.L.)

**Keywords:** *DICER1*, *DROSHA*, genetics, glaucoma, intraocular pressure, microRNA, polymorphisms, pseudoexfoliation, rs10719, Saudi

## Abstract

We investigated the association between *DICER1* (rs3742330) and *DROSHA* (rs10719) polymorphisms and pseudoexfoliation glaucoma (PXG) and related clinical phenotypes in a Saudi cohort. In a retrospective case-control study, TaqMan real-time, PCR-based genotyping was performed in 340 participants with 246 controls and 94 PXG cases. The minor (G) allele frequency of rs3742330 in PXG (0.03) was significantly different from that in the controls (0.08) and protective against PXG (odds ratio (OR) = 0.38, 95% confidence interval (CI) = 0.16–0.92), *p* = 0.017). Similarly, the rs3742330 genotypes showed a significant protective association with PXG in dominant (*p* = 0.019, OR = 0.38, 95% CI = 0.15–0.92), over-dominant (*p* = 0.024, OR = 0.39, 95% CI = 0.16–0.95), and log-additive models (*p* = 0.017, OR = 0.38, 95% CI = 0.16–0.92). However, none remained significant after an adjustment for age, sex, and multiple testing. Rs10719 in DROSHA did not show any significant allelic or genotype association with PXG. However, a protective effect of the GA haplotype in *DICER1* and *DROSHA* and PXG (*p* = 0.034) was observed. Both polymorphisms showed no significant effect on intraocular pressure and the cup–disk ratio. In conclusion, we report a significant genetic association between variant rs3742330 in *DICER1*, a gene involved in miRNA biogenesis, and PXG. Further investigation in a larger group of patients of different ethnicities and functional studies are warranted to replicate and validate its potential role in PXG.

## 1. Introduction

Pseudoexfoliation glaucoma (PXG) is an age-related and more aggressive form of open-angle glaucoma associated with a poor prognosis. PXG is characterized by excessive production and abnormal accumulation or deposition of pseudoexfoliative material, typically in the anterior segment of the eye that obstructs the aqueous flow pathway, leading to increased intraocular pressure (IOP), optic nerve head damage, retinal ganglion cell death (RGC), and subsequent loss of vision [1,2]. Genetic and environmental factors also play an important role in the development and progression of the disease [3,4]. Previous genome-wide studies have identified genetic loci and polymorphisms associated with the disease phenotype [3,5,6]. However, the genetic factors and molecular mechanisms contributing to glaucomatous eye damage are still unclear.

Increasing evidence suggests the critical roles of microRNA (miRNA) in glaucoma [7,8]. miRNAs are small (~22 bp), conserved, noncoding RNAs that act by binding to complementary sequences in the 3′ untranslated region (3′ UTR) of messenger RNAs (mRNAs) to regulate posttranscriptional gene expression or translational repression [9]. miRNAs regulate nearly 30% of human genes and each miRNA can regulate several gene targets [10]. Consequently, miRNAs can influence various pathophysiological processes, such as proliferation, differentiation, migration, and apoptosis, and modulate several disease outcomes as a result [11,12]. Two critical RNase III enzymes, DROSHA and DICER1, are involved in the biogenesis of miRNAs. Following the synthesis of primary miRNAs (pri-miRNAs) by RNase II in the nucleus, DROSHA cleaves pri-miRNAs into a 70 bp stem-loop structure called pre-miRNA. Subsequently, pre-miRNA is transported by the exportin-5 protein to the cytoplasm, wherein DICER1 processes pre-miRNAs into mature miRNAs that are subsequently incorporated into the RNA-induced silencing complex (RISC) to interact with target mRNAs and regulate their expression and function [13].

Differential expression of DICER1 or DROSHA enzymes due to polymorphisms in the miRNA coding genes can have pathological consequences [14,15]. Two commonly investigated variants affecting the miRNA binding and mRNA stability, expression, and function include rs10719 G > A in *DROSHA* and rs3742330 A > G in *DICER1*, located in the 3′ UTRs of their respective genes. These polymorphisms have been associated with several complex human diseases, including glaucoma [7,16,17,18,19]. We hypothesize that these polymorphisms via miRNA regulation of mRNA stability or translational repression might trigger downstream changes to influence disease processes (e.g., extracellular matrix (ECM) remodeling and trabecular meshwork (TM) homeostasis) involved in PXG pathogenesis [20]. Thus, we investigated the genetic association of polymorphisms rs3742330 and rs10719 in *DICER1* and *DROSHA* genes, respectively, in PXG patients of Saudi origin.

## 2. Materials and Methods

### 2.1. Study Design and Participants

In a retrospective case-control study, the participants were recruited at the King Abdulaziz University Hospital in Riyadh, Saudi Arabia. PXG patients (*n* = 94) of Saudi origin were diagnosed by glaucoma consultants based on the clinical evidence of exfoliation material on the pupil margin or anterior lens surface, the presence of glaucomatous optic neuropathy with associated visual field loss in one or both eyes, and high untreated IOP (≥21 mm Hg) as per the guidelines of the European Glaucoma Society [21]. The exclusion criteria included secondary glaucoma, optic neuropathies or visual loss unrelated to glaucoma, inability to examine fundus for disk assessment, steroid usage, ocular trauma, and refusal to participate. A group of healthy subjects (*n* = 246) of the same ethnicity, who were >40 years of age, had normal IOP (without medication), and were free from any form of glaucoma, was included as a control group. Subjects refusing to participate were excluded.

### 2.2. Genotyping of rs3742330 in DICER1 and rs10719 in DROSHA

DNA extracted from EDTA blood using a QIAamp DNA blood kit (Cat. # 51306, QIAGEN GmbH, Hilden, Germany) was subjected to genotyping. TaqMan^®^ assays C__27475447_10 and C___7761648_10 (Catalog number: 4351379, Applied Biosystems Inc., Foster City, CA, USA) were used to genotype rs3742330 (A>G) and rs10719 (G>A), respectively, under the recommended conditions on an ABI 7500 Real-Time PCR system (Applied Biosystems Inc., Foster City, CA, USA). Briefly, each PCR reaction was performed in a total volume of 25 µL consisting of a 1× TaqMan^®^ genotyping master mix (Applied Biosystems Inc., Foster City, CA, USA), 1X SNP genotyping assay mix, and 20 ng DNA. Each 96-well plate included two no-template (negative) controls. Amplification conditions included incubation at 95 °C for 10 min, followed by 40 cycles, denaturation at 92 °C for 15 s, and annealing and extension at 60 °C for 1 min. The VIC^®^ and 6-carboxy-fluorescein (FAM) fluorescence levels of the PCR products were measured at 60 °C for 1 min. A fluorescence analysis was performed using automated 2-color allele discrimination software to identify the *DICER1* and *DROSHA* genotypes on a 2-dimensional graph [22].

### 2.3. In Silico Analysis of rs3742330

The annotation of rs3742330 and its localization to the genomic region was visualized using the UCSC genome browser with the SNPedia option. Regulation and comparative genomics tracks were used to visualize regulatory elements and conservation across species. TargetScanHuman 7.2 was added as a custom track to visualize miRNA targets. The RNA binding partners to the 3′ UTR reference of 30 nucleotides upstream and downstream of rs3742330 were predicted using an RBPmap database (http://rbpmap.technion.ac.il/index.html, accessed on 24 February 2022). Regulatory motif alteration was examined using HaploReg v4.1.

### 2.4. Statistics

The Hardy–Weinberg equilibrium (HWE) and other allelic and genotype associations were tested by chi-square analysis. The continuous parameters were tested by an independent samples *t*-test and one-way ANOVA. Regression analysis was performed to evaluate the effect of multiple risk factors such as age, sex, and genotype on PXG outcome. Statistical analysis was performed using SPSS version 22 (IBM Inc., Chicago, IL, USA) and SNPStats online software (https://www.snpstats.net/start.htm, accessed on 30 December 2021). SHEsis online software (Analysis tool for random samples, by YongYong Shi (analysis.bio-x.cn, accessed on 30 December 2021)) was used to examine linkage disequilibrium (LD) and analyze haplotypes. Power analysis was performed using PS software (https://ps-power-and-sample-size-calculation.software.informer.com/3.1/, accessed on 30 December 2021). A two-tailed *p* < 0.05 was considered statistically significant and a Bonferroni-corrected *p*-value (0.05/5 = 0.01) was considered where applicable.

## 3. Results

### 3.1. Demographic Characteristics and Minor Allele Frequency Distribution

Table 1 shows the demographic characteristics of the participants and minor allele frequency (MAF) distribution of the examined polymorphisms. The cases were significantly older than the controls (*p* < 0.001). However, the differences in sex distribution between PXG cases compared to controls were nonsignificant. Of 246 controls and 94 PXG cases, genotyping was available for 241 controls for rs3742330 *DICER1* polymorphism. The five DNA samples from controls that failed to amplify were excluded from *DICER1* analysis. There was no significant deviation from the HWE (*p* > 0.05). The rs3742330 (G) MAF in PXG (0.03) varied more significantly than the controls (0.08) and was protective against PXG (odds ratio (OR) = 0.38, 95% confidence interval (CI) = 0.16–0.92), *p* = 0.017). In contrast, the rs10719 (A) allele showed no significant distribution between PXG and the controls. Furthermore, no significant sex-specific allelic association was observed (Table 1).

### 3.2. Genotype Association Analysis with PXG

PXG is a complex disease with no apparent genetic mode of inheritance. We used co-dominant, dominant, recessive, over-dominant, and log-additive genetic models to examine the association between *DICER1* and *DROSHA* polymorphisms and the risk of PXG using SNPStat software (Table 2). Rs3742330 genotypes in *DICER1* showed a significant association with PXG risk in dominant, over-dominant, and log-additive genetic models. The log-additive model exhibited the best fit with the lowest Akaike information criterion (AIC) and Bayesian information criterion (BIC) values. However, the significance was lost after an adjustment for age, sex, and Bonferroni correction (*p*_correction_ < 0.01). In contrast, rs10719 polymorphism in *DROSHA* showed no significant association (Table 2). In addition, a sex-stratified genotype analysis for rs3742330 and rs10719 showed no significant association (Table 3 and Table 4).

### 3.3. Linkage and Haplotype Analysis

The two polymorphisms were tested for LD and haplotype analysis using the SHEsis platform. The standardized LD coefficient D’ value between rs3742330 and rs10719 was 0.06 and *r^2^* = 0.00, indicating that these polymorphisms are not in LD. Additionally, the haplotype distribution did not significantly affect the risk of PXG (*X^2^* = 6.357, *df* = 3, *p* = 0.095). However, haplotype GA showed a significant protective effect (*p* = 0.034, OR = 0.20, 95% CI = 0.04–1.03, Table 5).

### 3.4. Regression Analysis and Genotype Influence on Clinical Parameters

**Figure 1:**
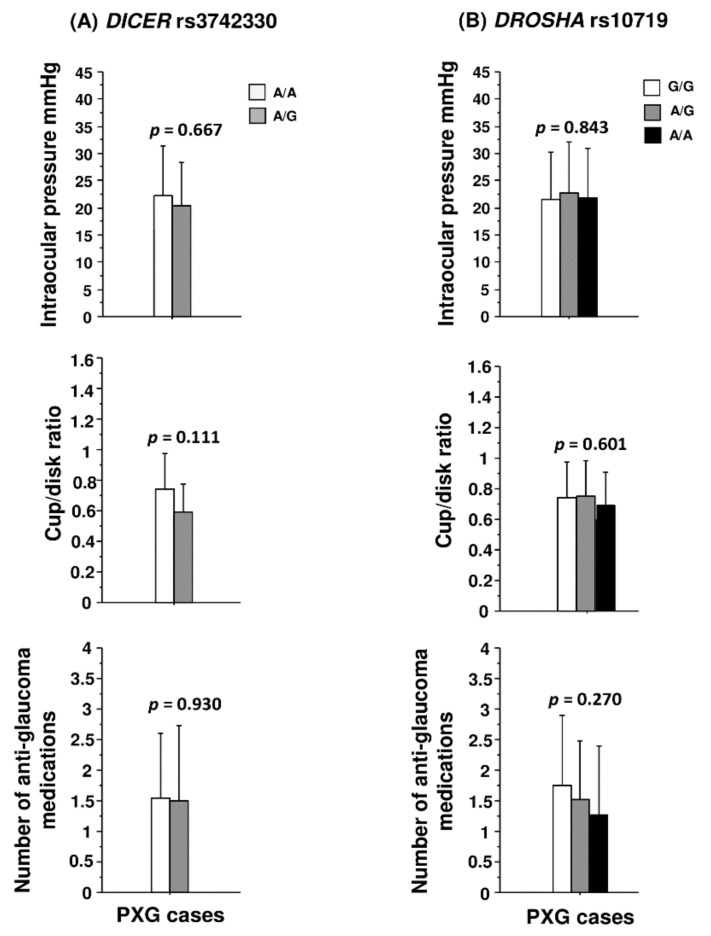
Genotype effect of (**A**) rs3742330 (*DICER1*) and (**B**) rs10719 (*DROSHA*) on glaucoma specific clinical indices in pseudoexfoliation glaucoma (PXG). Note: No rare homozygous rs3742330 G/G genotype was observed in pseudoexfoliation glaucoma cases. *p*-value is based on one-way ANOVA test.

A binary logistic regression analysis was performed to assess the influence of multiple risk factors such as age, sex, and genotypes of rs3742330 and rs10719 on PXG outcome. Except for age (*p* < 0.001), no other variable showed a significant contribution to PXG risk (Table 6). In addition, neither polymorphism showed a significant genotype effect on clinical markers such as IOP, the cup–disk ratio, and the number of antiglaucoma medications (Figure 1).

### 3.5. Potential Significance of rs3742330

The genomic region containing rs3742330 and its associated neighboring features is shown in Supplementary Figure S1. An in silico analysis showed that the region contains multiple sites for miRNAs, transcription factors, and other regulatory elements (Figure S1A). In addition, the variant may alter the binding of certain RNA-binding proteins (Figure S1B) and regulatory motifs (Figure S1C), suggesting that the region is significant for mRNA stability and gene expression. However, rs3742330 is classified as benign in ClinVar.

## 4. Discussion

In this paper, we report for the first time a moderate allelic association of variant rs3742330 (G) in *DICER1* with PXG in a Saudi cohort. The polymorphism rs3742330 in *DICER1* was associated with a decreased risk of PXG (OR of 0.38). Although no homozygous G/G genotype was observed in the PXG patients, the heterozygous A/G genotype also conferred significant protection compared to A/A in different genetic models. However, it did not survive correction for multiple testing.

Emerging evidence suggests that polymorphism(s) in *DICER1* may alter the biological functions of miRNAs and play an essential role in the pathogenesis of various diseases [16,17,18,19]. In accordance with our findings, Chatzikyriakidou et al. reported a different *DICER1* variant, rs1057035 (C>T), which conferred protection (OR of 0.69) in patients with pseudoexfoliation syndrome [7]. Using the LDlink analysis (https://ldlink.nci.nih.gov/?tab = ldpop, accessed on 30 December 2021) to predict linkage across the 1000 Genomes database, we noted that variants rs1057035 and rs3742330 in *DICER1* were not in LD (*r^2^* = 0.032); yet, this study lends further support to the protective association of the *DICER1* variant observed in our patient cohort.

*Dicer* has an essential role in development and angiogenesis [23,24]. Germ-line mutations in *DICER1* are associated with *DICER1* syndrome [25]. *DICER1* carriers exhibit ocular abnormalities including optic nerve damage and retinal changes [25]. Genetic manipulation of *dicer1* in mice has been shown to cause retinal degeneration, suggesting its role in cell survival [26,27]. Differential expressions of *DICER1* have been associated with various types and stages of cancers, albeit with contradictory findings. The increased expression has been linked to a good prognosis in lung, breast, and ovarian cancer as opposed to a poor prognosis in colorectal and prostate cancer [28,29,30]. These studies suggest that *DICER1* may have varied roles in different diseases. The underlying potential mechanism(s) linking *DICER1* polymorphism rs3742330 to PXG pathogenesis is unknown. Rs3742330 polymorphism has been related to dysregulation of *DICER1* mRNA, wherein the polymorphic A/G and G/G genotypes harbored lower levels of *DICER1* mRNA [31,32]. Altered DICER1 levels (due to polymorphism) may directly affect enzyme function or can indirectly influence disease pathogenesis via differential regulation of miRNA expression.

*Dicer* knockdown results in a marked global reduction in miRNA levels [33,34]. miRNAs are tissue-specific and expressed in ocular tissues related to glaucoma [35]. Several studies have highlighted the significant role of miRNAs and their plausible underlying mechanisms in different types of glaucoma, including PXG [8,36,37,38,39,40]. Rao et al. reported the overexpression of 12 miRNAs in PXG, of which miR-122-5p, miR-124-3p, and miR-424-5p targeted TGFβ1, fibrosis/ECM, and proteoglycan metabolism pathways [39]. These signaling pathways are known mediators of ECM secretion and deposition and are strongly implicated in glaucoma [41]. Interestingly, using MicroSNiPer [42], we observed that the *DICER1* rs3742330 (G) variant might enhance the binding of hsa-miR-124-3p and probably cause a decrease in *DICER1* gene expression, which may impact the DICER1 enzyme’s function and/or sequentially affect miRNA expression. Similarly, Zenkel et al. reported downregulation of the miR-29 family in iridal and ciliary tissue specimens from donor eyes with pseudoexfoliation syndrome [43]. In vitro studies have reported the miR-29 family as an essential modulator of ECM genes under chronic oxidative stress conditions and TGFß stimulation in the human TM cells [44,45]. miR-1260b, mainly involved in the process of hypoxia and apoptosis, was reported to be most abundantly expressed in PXG [38]. Hindle et al. reported 20 circulatory miRNAs targeting neuroinflammation, nitric oxide, and neurotrophin–tropomyosin-related kinase (TRK) signaling pathways [40], which have all been strongly implicated in the pathophysiology of glaucoma [46,47]. In another study, Drewry et al. identified dysregulated miRNAs (miR-122-5p, miR-3144-3p, miR-320a, miR-320e, and miR-630) in PXG involved in focal adhesion, tight junctions, and TGFß signaling [37].

Oxidative stress, inflammation, breakdown of the blood–aqueous barrier, a decrease in clusterin, and genetic predisposition (e.g., lysyl oxidase-like-1 (*LOXL1*) polymorphisms) are among the pathologic mediators of the abnormal elastotic process in PXG [1]. Taken together, we speculate that polymorphism rs3742330 in *DICER1* may alter DICER levels and, via miRNA regulation, may be associated with PXG by influencing processes or pathways discussed above (e.g., TRK signaling, TGFß signaling, oxidative stress genes, tight junctions, and apoptosis) that might affect ECM remodeling and TM homeostasis or RGC survival. We are interested in discovering whether the *DICER1* polymorphism modulates the PXG risk by affecting DICER enzyme function and/or via RNA interference. The in silico analysis suggested that the genomic region of rs3742330 located in the 3′ UTR of *DICER1* may be important for mRNA transcript stability and post-transcriptional regulation of gene expression. However, there is no direct evidence supporting this role. Additionally, the effect of gene–gene and/or gene–environment interactions, or linkage with a causal variant in PXG, cannot be ruled out. Further molecular and in vitro studies are needed to validate these hypotheses.

In contrast, rs10719 polymorphism in *DROSHA*, another critical enzyme involved in miRNA biogenesis [33], did not show any allelic or genotype association with PXG or clinical markers (e.g., IOP and the cup–disk ratio), indicating that this variant may not have a significant role in PXG. However, the role of other variants in this gene cannot be ruled out. In addition, haplotype analysis of *DICER1* and *DROSHA* polymorphisms indicated that haplotype GA was protective against PXG. However, it is difficult to ascertain whether the protective effect observed in our study is attributable to a real haplotype effect or reflects a strong LD with any other variant(s) not included in this study.

The results of the study have certain limitations. It should be noted that the significant association of the *DICER1* variant was restricted to the unadjusted models of analysis when corrections for multiple testing and confounding variables were included, indicating that the association may be dependent on other gene–gene or gene–environmental interactions [48]. It also needs to be emphasized that the study was exploratory, performed in a single ethnic group of patients, and provides no functional evidence. In addition, the results are limited by sample size, particularly considering the low frequency of the variant observed in this cohort of Arab ethnicity, and the possibility of chance association cannot be ruled out. Based on the observed allele frequencies and an α level of 0.05, the estimated power was 0.73 per allele for rs3742330 (*DICER1*) and >0.9 per allele for rs10719 (*DROSHA*) to detect an OR of two. However, a much larger sample size would be required to detect an odds ratio of 1.5 or less, which is commonly seen in genetic association studies.

In conclusion, we report a genetic association between a potentially functional polymorphism rs3742330 in *DICER1*, a gene involved in miRNA biogenesis, and PXG patients of Saudi origin, suggesting a need to further investigate the involvement of epigenetic pathways as modulators in disease development and progression. Further replication in a large population-based sample cohort of different ethnicities and functional studies are warranted to validate the potential role of *DICER1* variant rs3742330 in PXG.

## Figures and Tables

**Table 1:** Demographic characteristics and distribution of minor allele frequency of *DICER1* rs3742330 (G) and *DROSHA* rs10719 (A) polymorphisms in pseudoexfoliation glaucoma cases and control participants.

Characteristics	Controls	Cases	Odds Ratio (95% Confidence Interval)	*p*-Value
No. of participants	246	94	-	-
Age in years (SD)	59.5 (7.2)	66.4 (9.7)	-	<0.001 ^1^
Men, *n* (%)	132 (53.6)	61 (64.8)	-	-
Women, *n* (%)	114 (46.3)	33 (33.5)	-	0.061 ^2^
**Minor Allele Frequency**				
rs3742330 (G)				
Total	0.08	0.03	**0.38 (0.16–0.92)**	**0.017** **^2^**
Men	0.08	0.03	0.40 (0.13–1.21)	0.076 ^2^
Women	0.08	0.03	0.34 (0.07–1.55)	0.120 ^2^
rs10719 (A)				
Total	0.43	0.45	1.08 (0.78–1.50)	0.630 ^2^
Men	0.45	0.47	1.08 (0.71–1.65)	0.720 ^2^
Women	0.41	0.42	1.05 (0.62–1.77)	0.870 ^2^

^1^ Independent *t*-test; ^2^ chi-square analysis. Significant odds ratio and *p*-value in bold.

**Table 2:** Association analysis of polymorphisms rs3742330 in *DICER1* and rs10719 in *DROSHA* with the risk of pseudoexfoliation glaucoma cases compared to controls under different genetic models.

SNP Number	Genetic Model ^1^	Genotype	Controls *n* (%)	Cases *n* (%)	Odds Ratio (95% Confidence Interval)	*p*-Value ^2^	AIC ^3^	BIC ^4^	*p*-Value ^2,5^
rs3742330	Co-dominant	A/A	204 (84.7)	88 (93.6)	1.00	0.055	397.9	409.3	0.170
		A/G	36 (14.9)	6 (6.4)	**0.39 (0.16–0.95)** ^6^				
		G/G	1 (0.4)	0 (0.0)	0.00 (0.00-NA)				
	Dominant	A/A	204 (84.7)	88 (93.6)	1.00	**0.019**	396.2	403.8	0.077
		A/G-G/G	37 (15.3)	6 (6.4)	**0.38 (0.15–0.92)**				
	Recessive	A/A-A/G	240 (99.6)	94 (100.0)	1.00	0.420	401.0	408.6	0.380
		G/G	1 (0.4)	0 (0.0)	0.00 (0.00-NA)				
	Over-dominant	A/A-G/G	205 (85.1)	88 (93.6)	1.00	**0.024**	396.6	404.2	0.098
		A/G	36 (14.9)	6 (6.4)	**0.39 (0.16–0.95)**				
	Log-additive	-	-	-	**0.38 (0.16–0.92)** ^7^	**0.017**	396.0	403.6	0.068
rs10719	Co-dominant	G/G	82 (33.3)	32 (34.0)	1.00	0.530	405.6	417.1	0.250
		A/G	116 (47.1)	39 (41.5)	0.86 (0.50–1.49)				
		A/A	48 (19.5)	23 (24.5)	1.23 (0.65–2.34)				
	Dominant	G/G	82 (33.3)	32 (34.0)	1.00	0.900	404.9	412.6	0.350
		A/G-A/A	164 (66.7)	62 (66.0)	0.97 (0.59–1.60)				
	Recessive	G/G-A/G	198 (80.5)	71 (75.5)	1.00	0.320	403.9	411.6	0.350
		A/A	48 (19.5)	23 (24.5)	1.34 (0.76–2.35)				
	Over-dominant	G/G-A/A	130 (52.9)	55 (58.5)	1.00	0.350	404.0	411.7	0.098
		A/G	116 (47.1)	39 (41.5)	0.79 (0.49–1.29)				
	Log-additive	-	-	-	1.08 (0.78–1.50)	0.630	404.7	412.3	0.930

^1^ Tested by SNPStat; ^2^ chi-square analysis; ^3^ AIC, Akaike information criterion; ^4^ BIC, Bayesian information criterion; ^5^ adjusted for age and sex; ^6^ A/A vs. A/G *p*-value = 0.032; ^7^ best-fit model *p*-value. Significant odds ratio and *p*-value in bold. Bonferroni-corrected *p*-value is 0.01.

**Table 3:** Association analysis of rs3742330 polymorphism in *DICER1* with pseudoexfoliation glaucoma cases in men and women.

Group	Genetic Model ^1^	Genotype	Control *n* (%)	Cases *n* (%)	Odds Ratio (95% Confidence Interval)	*p*-Value ^2^	AIC ^3^	BIC ^4^	*p*-Value ^2,5^
Men	Co-dominant	A/A	109 (85.2)	57 (93.4)	1.00	0.190	240.4	250.1	0.430
		A/G	18 (14.1)	4 (6.6)	0.42 (0.14–1.32)				
		G/G	1 (0.8)	0 (0.0)	0.00 (0.00–NA)				
	Dominant	A/A	109 (85.2)	57 (93.4)	1.00	0.087	238.8	245.3	0.280
		A/G-G/G	19 (14.8)	4 (6.6)	0.40 (0.13–1.24)				
	Recessive	A/A-A/G	127 (99.2)	61 (100.0)	1.00	0.380	241.0	247.4	0.380
		G/G	1 (0.8)	0 (0.0)	0.00 (0.00–NA)				
	Over-dominant	A/A-G/G	110 (85.9)	57 (93.4)	1.00	0.120	239.3	245.7	0.350
		A/G	18 (14.1)	4 (6.6)	0.43 (0.14–1.33)				
	Log-additive	-	-	-	0.40 (0.13–1.21)	0.076	238.6	245.1	0.240
Women	--	A/A	95 (84.1)	31 (93.9)	1.00	0.120	157.6	163.6	0.120
		A/G	18 (15.9)	2 (6.1)	0.34 (0.07–1.55)				
		G/G	0 (0.0)	0 (0.0)	-				

^1^ Tested by SNPStat; ^2^ chi-square analysis; ^3^ AIC, Akaike information criterion; ^4^ BIC, Bayesian information criterion; ^5^ adjusted for age and sex. Note: No rare homozygous genotype G/G was observed among women.

**Table 4:** Sex-stratified association analysis of polymorphism rs10719 in *DROSHA* with pseudoexfoliation glaucoma cases.

Group	Genetic Model ^1^	Genotype	Control *n* (%)	Cases *n* (%)	Odds Ratio (95% Confidence Interval)	*p* ^2^	AIC ^3^	BIC ^4^	*p* ^2,5^
Men	Co-dominant	G/G	40 (30.3)	20 (32.8)	1.00	0.450	245.2	255.0	0.230
		A/G	66 (50)	25 (41.0)	0.76 (0.37–1.54)				
		A/A	26 (19.7)	16 (26.2)	1.23 (0.54–2.80)				
	Dominant	G/G	40 (30.3)	20 (32.8)	1.00	0.730	244.7	251.2	0.470
		A/G-A/A	92 (69.7)	41 (67.2)	0.89 (0.46–1.71)				
	Recessive	G/G-A/G	106 (80.3)	45 (73.8)	1.00	0.310	243.8	250.3	0.230
		A/A	26 (19.7)	16 (26.2)	1.45 (0.71–2.96)				
	Over-dominant	G/G-A/A	66 (50.0)	36 (59.0)	1.00	0.240	243.4	250.0	0.094
		A/G	66 (50.0)	25 (41.0)	0.69 (0.38–1.28)				
	Log-additive	-	-	-	1.08 (0.71–1.65)	0.720	244.7	251.2	0.830
Women	Co-dominant	G/G	42 (36.8)	12 (36.4)	1.00	0.970	162.5	171.5	0.800
		A/G	50 (43.9)	14 (42.4)	0.98 (0.41–2.35)				
		A/A	22 (19.3)	7 (21.2)	1.11 (0.38–3.23)				
	Dominant	G/G	42 (36.8)	12 (36.4)	1.00	0.960	160.6	166.5	0.510
		A/G-A/A	72 (63.2)	21 (63.6)	1.02 (0.46–2.28)				
	Recessive	G/G-A/G	92 (80.7)	26 (78.8)	1.00	0.810	160.5	166.5	0.900
		A/A	22 (19.3)	7 (21.2)	1.13 (0.43–2.93)				
	Over-dominant	G/G-A/A	64 (56.1)	19 (57.6)	1.00	0.8800	160.5	166.5	0.590
		A/G	50 (43.9)	14 (42.4)	0.94 (0.43–2.06)				
	Log-additive	-	-	-	1.05 (0.62–1.77)	0.870	160.5	166.5	0.610

^1^ Tested by SNPStat; ^2^ chi-square analysis; ^3^ AIC, Akaike information criterion; ^4^ BIC, Bayesian information criterion; ^5^ adjusted for age and sex.

**Table 5:** Haplotype analysis of DICER1 rs3742330 and DROSHA rs10719 polymorphisms.

Haplotypes ^1^	Controls, Frequency	Cases, Frequency	*p*-Value	Odds Ratio (95% Confidence Interval)
AA	0.38	0.44	0.179	1.26 (0.89–1.77)
AG	0.53	0.52	0.821	0.96 (0.68–1.35)
GA	0.04	0.01	**0.034**	**0.20 (0.04–1.03)**
GG	0.04	0.02	0.334	0.59 (0.21–1.71)

^1^ Tested by SHEsis in the order of rs3742330 and rs10719. Overall chi-square = 6.357, df = 3, *p* = 0.095. Significant *p*-value and odds ratio in bold.

**Table 6:** Binary logistic regression analysis to determine the effect of age, sex, and polymorphisms rs3742330 (*DICER1*) and rs10719 (*DROSHA*) on the risk of pseudoexfoliation glaucoma.

Group Variables	B	SE	Wald	Odds Ratio (95% Confidence Interval)	*p*
Age	0.100	0.017	35.755	1.10 (1.07–1.14)	**0.000**
Sex	0.392	0.273	2.068	1.48 (0.86–2.52)	0.150
rs3742330			2.350		0.309
A/G	−0.729	0.476	2.349	0.48 (0.19–1.22)	0.125
G/G	-	-	-	-	1.000
rs10719			3.002		0.223
G/A	−0.410	0.305	1.801	0.66 (0.36–1.20)	0.180
A/A	0.127	0.364	0.121	1.13 (0.55–2.31)	0.728
Constant	−7.236	1.079	44.981	0.001	0.000

Significant *p*-value in bold.

## Data Availability

The data supporting the conclusions of this article are all presented within the report.

## Supplementary Materials

The following supporting information can be downloaded at: https://www.mdpi.com/article/10.3390/genes13030489/s1, Figure S1: The in silico analysis of rs3742330 in DICER1 showing (A) The genomic region containing the polymorphism on chromosome 14q32.13 and its neighboring features, such as miRNA binding sites, transcription binding sites, regulatory elements, eQTLs, and conservation among species obtained using the UCSC genome browser. (B) The altered RNA binding protein partners predicted using RBP map database. (C) Altered regulatory motifs as detected by HaploReg v4.1.
