# Supplementary material for: Polymorphism rs3742330 in microRNA Biogenesis Gene DICER1 Is Associated with Pseudoexfoliation Glaucoma in Saudi Cohort
Source: Genes (Basel). 2022 Mar 10;13(3):489. doi: 10.3390/genes13030489 (PMC8956095; doi:10.3390/genes13030489)
Supplement: Supplementary file 1 [file genes-13-00489-s001.zip › genes-1604003-supplementary.pdf]

(A)

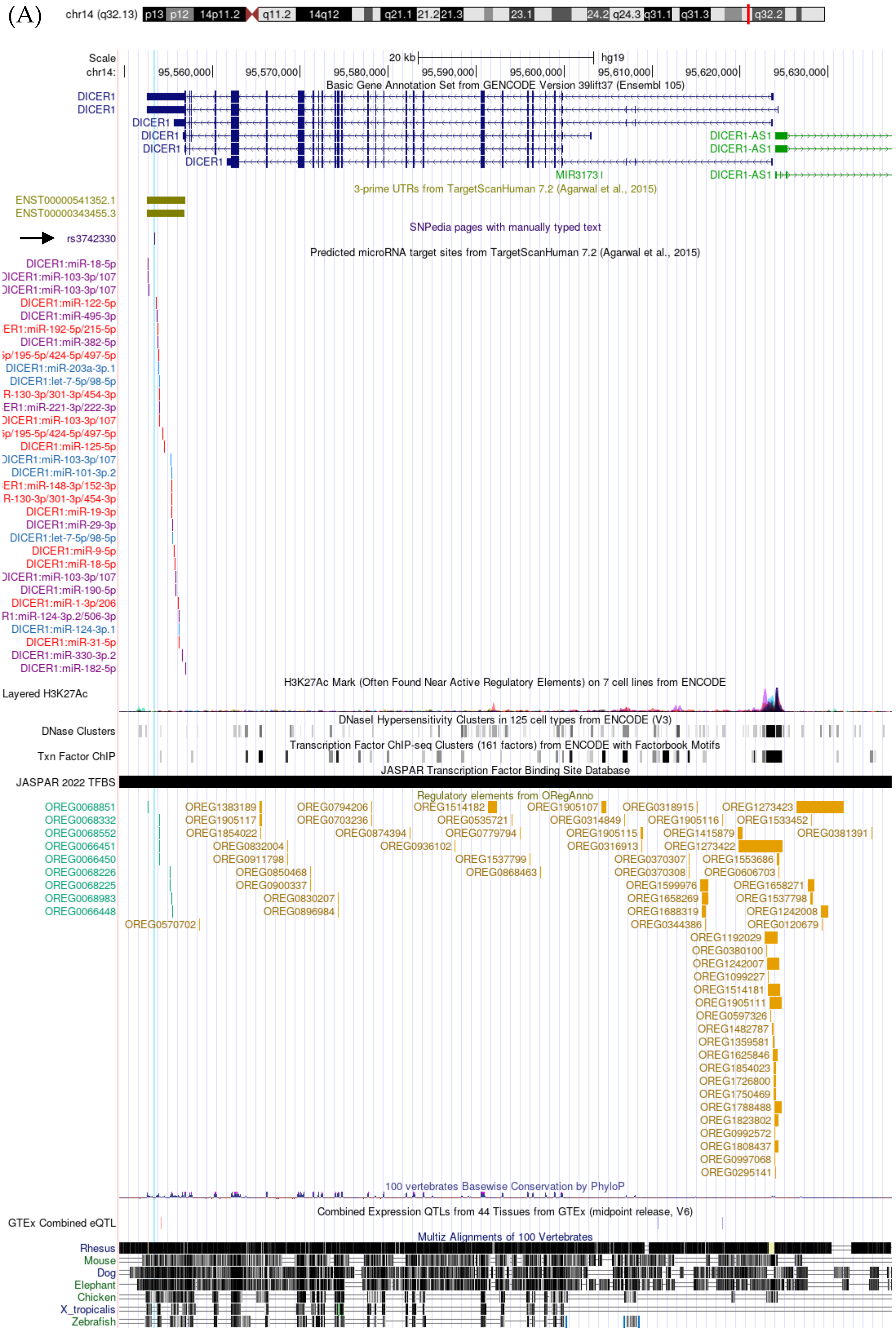

(B)

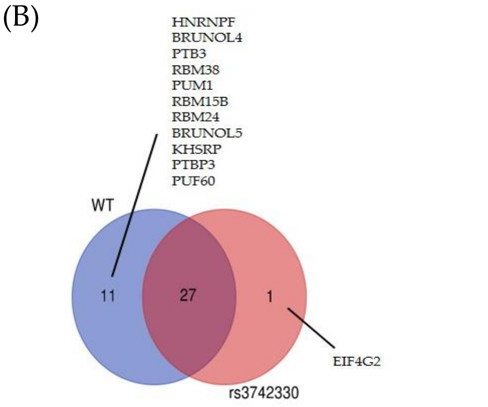

The binding partners to the 3'UTR reference to 30 nucleotide upstream and downstream of rs3742330 used to predict, the motifs from RBPmap database http://rbpmap.technion.ac.il/index.html. Human / Mouse motifs was used as option run the prediction.

(C)

Regulatory motifs altered

Position Weight Matrix ID	Strand	Ref	Alt	Match on:<br>Ref:<br>GTGGCTTCAATCTTGTGTAAAGGGATTAGACACCCTAACAGAGCAAGATCCAATATTTT<br>Alt:<br>GTGGCTTCAATCTTGTGTAAAGGGATTAGGCACCCTAACAGAGCAAGATCCAATATTTT
Crx_1	+	11.1	11.4	VKVRGATTARDVR
Crx_2	+	11.4	11.5	YBWDGSGGATTADCSD
Dmbx1	+	10.9	12.9	WDRWNVGGATTADKBNW
Gsc	+	12.9	13.3	BDHRSGGATTARYDWNW
Obox6	+	12	9.7	MNAWDSGGATTAWHB
Otx2	+	13.4	13.5	NDDRRGGATTAATHDNH
Pitx3	+	12	13.1	VRSRGGATTADCKRBS
Smad_1	+	10.5	-1.2	AGACWBNNN

Figure S1: The *in silico* analysis of rs3742330 in *DICER1* showing (A) The genomic region containing the polymorphism on chromosome 14q32.13 and its neighboring features, such as miRNA binding sites, transcription binding sites, regulatory elements, eQTLs, and conservation among species obtained using the UCSC genome browser. (B) The altered RNA binding protein partners predicted using RBP map database. (C) Altered regulatory motifs as detected by HaploReg v4.1.
